# Importance of Habitat Context in Modelling Risk Maps for Two Established Invasive Alien Plant Species: The Case of *Ailanthus altissima* and *Phytolacca americana* in Slovenia (Europe)

**DOI:** 10.3390/plants13060883

**Published:** 2024-03-19

**Authors:** Maarten de Groot, Erika Kozamernik, Janez Kermavnar, Marija Kolšek, Aleksander Marinšek, Andreja Nève Repe, Lado Kutnar

**Affiliations:** 1 Slovenian Forestry Institute, Večna pot 2, 1000 Ljubljana, Slovenia; erika.kozamernik@gozdis.si (E.K.); janez.kermavnar@gozdis.si (J.K.); aleksander.marinsek@gozdis.si (A.M.); lado.kutnar@gozdis.si (L.K.); 2 Slovenia Forest Service, Večna pot 2, 1000 Ljubljana, Slovenia; marija.kolsek@zgs.si (M.K.); andreja.repe@zgs.si (A.N.R.)

**Keywords:** American pokeweed, tree of heaven, species distribution modelling, forests, forest disturbance, habitat suitability

## Abstract

Forests are important ecosystems that face threats from climate change and global environmental shifts, with invasive alien plant species being a significant concern. Some of these invasive species have already become established, while others are in the process of naturalisation. Although forests are a relatively stable ecosystem, extreme weather events increase their vulnerability to change, and clearings left after natural disturbances are particularly susceptible to invasion by alien plant species (IAPS). We created risk maps of two species that have spread rapidly in the last decade: American pokeweed (*Phytolacca americana*) and the tree of heaven (*Ailanthus altissima*). We prepared a generalised linear model based on the occurrence data collected within the LIFE ARTEMIS project. Eleven environmental variables were used to determine habitat characteristics. We constructed two models for each species: one covering the entirety of Slovenia and the other specifically for the forested areas in Slovenia, with the latter incorporating forest-specific variables (such as forest sanitation felling and monocultures). We observed the presence of both species at lower altitudes and in close proximity to water sources. American pokeweed tends to occur nearer to railways, while the presence of the tree of heaven is associated with areas lacking carbonate parent material and influenced by land use patterns. In forested areas, the occurrence of American pokeweed is influenced by forest habitat characteristics, such as disturbances caused by extreme weather events or the prevalence of Norway spruce monocultures. In contrast, the occurrence of the tree of heaven is influenced by more general environmental variables, such as altitude and proximity to railways. Consequently, we have generated risk maps for the entirety of Slovenia and separately for forested areas, both of which indicate similar levels of risk, particularly for the tree of heaven. The risk map for American pokeweed highlights numerous vulnerable areas, especially forest edges, which are highly susceptible to invasion. Furthermore, there is a higher likelihood of this species occurring in areas that have undergone sanitation felling. This study suggests that the production of risk maps of IAPS could be improved by focussing on habitat types and taking into account habitat-specific variables. This approach could enhance the early detection and management of these invasive species.

## 1. Introduction

The presence of invasive alien species in forests has a profound impact on ecosystem services [1], as it negatively impacts biodiversity, the economy and human health. In recent decades, alien species have expanded exponentially, and this trend appears to be continuing [2]. Only 15% of non-native species actually become invasive in the sense that they affect biodiversity [3]. These species can significantly disrupt ecosystems through the competitive exclusion of native species, feeding and parasitising on plants or animals, interbreeding with native species, altering the environment, transmitting diseases and engaging in many other direct and indirect interactions [1,4]. Given these impacts, it is imperative to eradicate or, at the very least, control populations of invasive alien species to prevent their further spread.

The most effective strategy for halting the introduction of potentially invasive alien plant species is to eradicate them as soon as possible within the framework of an early detection and rapid response system [5,6]. In the case of *A. altissima,* it is even mandatory under the IAS EU regulation 1143/2014. However, this approach presents several challenges (e.g., public opinion, determining eradication zones, limited knowledge, inadequate funding), which can hinder efforts to prevent the spread of potentially invasive alien species [7,8,9]. Species that are already established and in the process of uncontrolled expansion pose an even greater threat. In such cases, it is essential to identify two types of priority and vulnerability areas: areas where invasive species are already thriving, and areas where these species are not yet present but have a high likelihood of becoming established. It is assumed that the ecological and other characteristics of the species make their occurrence in these areas highly probable. Special attention should be paid to controlling or eradicating these invasive alien species in these areas. To identify priority areas, it is necessary to create risk maps for the selected species, revealing the areas where the species already occurs or is most likely to occur in the future [10,11,12].

Forests are ecosystems with high biodiversity and provide many ecosystem services [13]. In general, forests exhibit a high resistance against invasive alien species from certain taxonomic groups, such as plants [14]. However, in recent decades, forests have faced increasing pressure from climate change [15], especially with the rising occurrence of large-scale disturbances [16] caused by windthrows, ice storms and pest outbreaks such as bark beetles [16,17,18,19]. Sanitary felling is often carried out after large-scale disturbances, potentially leaving the forest open and susceptible to invasive alien species. To prevent their spread in forests, it is crucial to understand whether forest openness resulting from disturbance and other management actions promotes the occurrence of these species.

In this study, we developed risk maps for two selected invasive alien species: the tree of heaven (*Ailanthus altissima* (Mill.) Swingle) and American pokeweed (*Phytolacca americana* L.). Predicting the potential habitat of high-risk invasive species can provide a scientific basis for quarantine and control strategies [20]. The tree of heaven is a deciduous tree found naturally in the northeastern and central parts of China and Taiwan [21,22]. It was introduced to America and Europe in the 18th century. It is considered one of the worst invasive plant species in Europe and is also listed as invasive in North America and many other countries [21]. This species is aggressive and directly and indirectly threatens other plant species. It is on the list of IAS of Union concern published under the EU regulation on invasive alien species (Regulation (EU) No 1143/2014 of the Euro-pean Parliament and of the Council of 22 October 2014 on the prevention and management of the introduction and spread of invasive alien species). Although it is widespread in Slovenia and occurs in various habitats from urban areas to grasslands and forests, it is most commonly found in open non-forested areas [23,24]. It primarily spreads through seeds, aided by wind and water, as well as through the transport of soil and building materials. It is generally considered a pioneer species. Locally, it can spread vegetatively very rapidly. Based on a limited set of data, a map of habitat suitability was created for the western part of Slovenia [23], but such information is not yet available for other regions of Slovenia.

American pokeweed is a poisonous herbaceous perennial native to North America [25]. Distribution via birds is thought to account for the appearance of isolated plants in areas otherwise free of American pokeweed [26]. The seeds have a long viability period, capable of germinating after many years in the soil. Once established, it affects local biodiversity by competing with native plant species for nutrients and light [27] and impacting ground-dwelling organisms [28,29]. Its fruit (berries) are toxic to cattle and humans [30,31]. While American pokeweed has been present in Slovenia for more than a century, it only started to spread invasively in the last two decades [32]. Limited research has been conducted on this species and its habitat preferences, particularly because it is a relatively new invasive alien species.

Both the tree of heaven and American pokeweed are highly invasive, but they occupy different ecological niches and possess distinct biology and traits. Therefore, it is vital to understand how these species may infiltrate forests and where they are likely to invade. The aim of the study is to create risk maps for the invasive non-native tree species *A*. *altissima* and *P*. *americana* in Slovenia, in order to help managers in the control/eradication of the two species. We investigated the influence of forest disturbances on the occurrence of these two invasive alien species, which differ according to their habitat preferences. Based on environmental variables, we aim to prepare a probability map of the risk/occurrence of the selected species for the entirety of Slovenia and separately for forested areas.

## 2. Results

### 2.1. Phytolacca americana

The model for the entire territory of Slovenia showed that the most influential variables are altitude, distance to water bodies and distance to railways (Table 1). American pokeweed is more likely to be found at lower altitudes, in close proximity to water bodies and near railways. Other variables were also included in the model but did not have a significant influence. Upon validation, the model demonstrated acceptable discriminatory power, with an AUC of 0.77 (Table 2).

In the case of Slovenian forests, the model highlighted the importance of spruce monocultures, geology (presence of carbonate or absence of carbonate parent material), the occurrence of sanitary felling, altitude and proximity to water bodies and railways as the most influential variables (Table 1). American pokeweed is more likely to be found in monocultures of Norway spruce, areas with non-carbonate (siliceous) parent material, locations with a history of sanitary felling, lower altitudes and in close proximity to water bodies and railways. Other variables were also included in the model but did not exhibit a significant influence. The validation of the forest model also demonstrated acceptable discrimination, with an AUC of 0.73 (Table 2).

**Figure 1:**
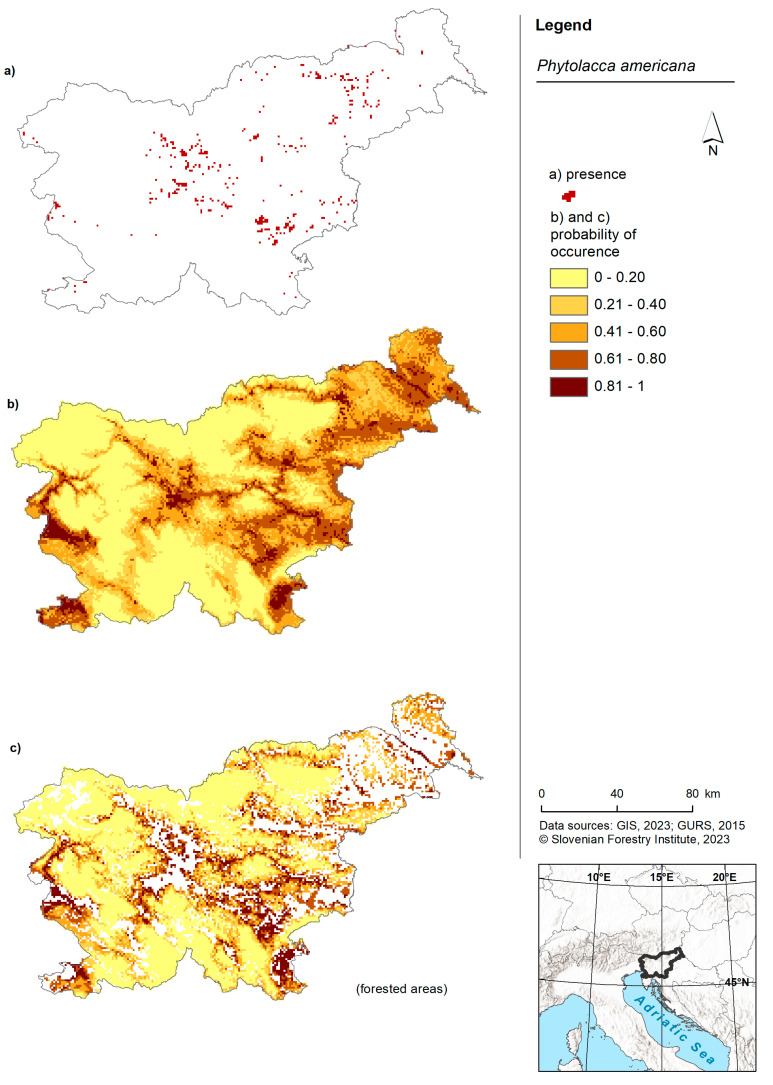
Risk maps of American pokeweed in Slovenia and for forested areas in Slovenia. (**a**) Shows the occurrence of American pokeweed in Slovenia as recorded in the Invazivke.si database; (**b**) shows a map displaying the probability of occurrence based on the model for the entire territory of Slovenia and (**c**) shows a map presenting the probability of occurrence based on the forest model within forested areas of Slovenia.

**Figure 2:**
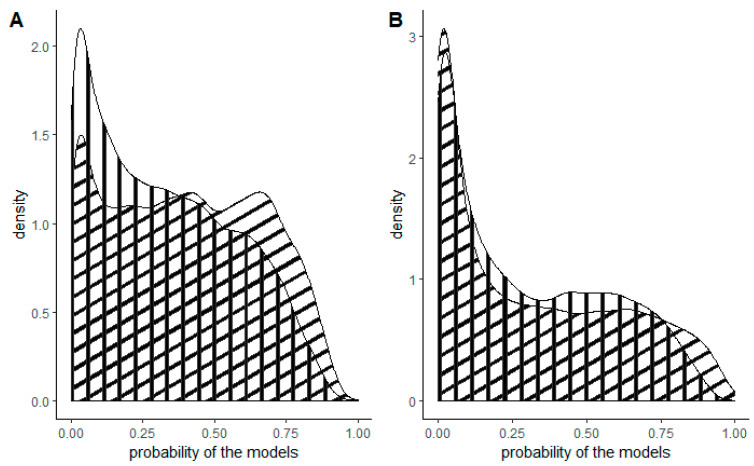
Density plots of the probability of the models for the entirety of Slovenia (vertical stripes) and forested areas only (diagonal stripes) for (**A**) tree of heaven and (**B**) American pokeweed.

American pokeweed was predicted to occur in the lowlands of Slovenia (Figure 1). Areas with the highest probability of occurrence were observed in the western part (near Nova Gorica), southern regions (around Koper) and the eastern part of the country. The forest model predicted the presence of American pokeweed in the western, southwestern, central and eastern regions of Slovenia. Once again, the highest probability was associated with lowland areas. While the model for American pokeweed across the entire territory of Slovenia was comparable to the forest model, the latter exhibited slightly more cells with a higher probability (Figure 2B).

### 2.2. Ailanthus altissima

The model for the entire territory of Slovenia revealed that the most important variables were altitude, insolation, land use and distance to water bodies (Table 1). Tree of heaven is more likely to be found in areas with non-carbonate bedrock, high insolation, lower altitudes and in close proximity to water bodies. Other variables were also included in the model but did not have a significant influence. Upon validation, the model demonstrated excellent discriminatory power, with an AUC of 0.82 (Table 2).

In the case of Slovenian forests, the model showed that the most important variables are altitude and distance to railways (Table 1). Tree of heaven is more likely to be found at lower altitudes and farther away from railways within forested areas. Other variables were also included in the model but did not have a significant influence. The validation of the forest model also demonstrated excellent discrimination, with an AUC of 0.83 (Table 2).

**Figure 3:**
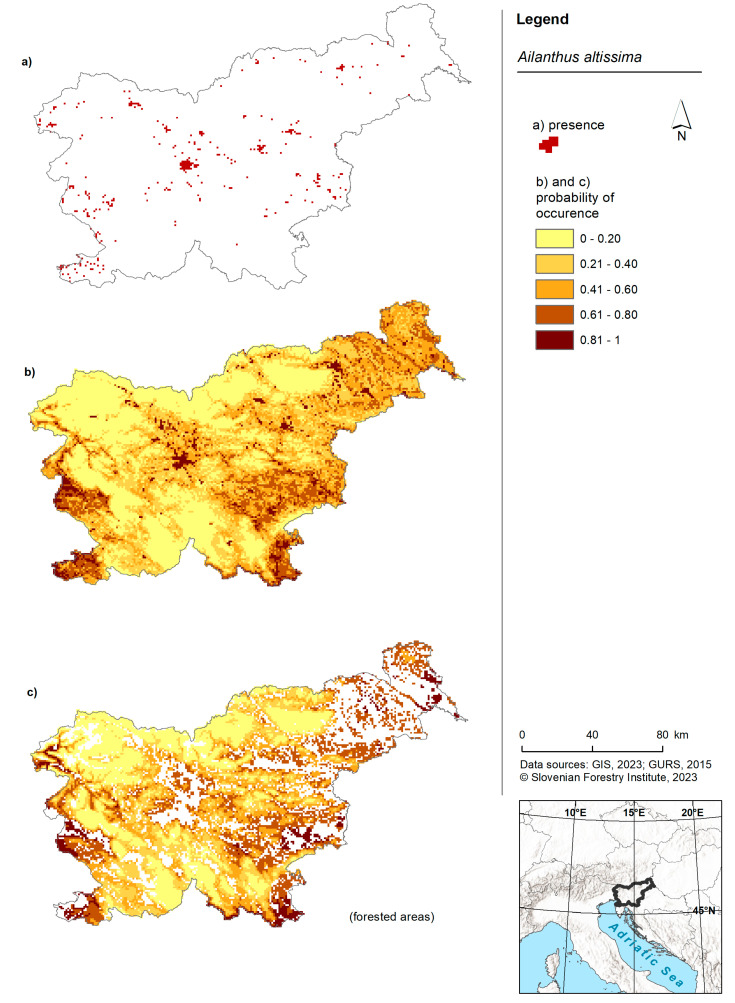
Risk maps of the tree of heaven in Slovenia and for forested areas. (**a**) Shows the occurrence of American pokeweed in Slovenia according to the Invazivke.si database; (**b**) shows a map of the probability of occurrence based on the model for the entire territory of Slovenia and (**c**) shows a map presenting the probability of occurrence based on the forest model within forested areas of Slovenia.

For the entire territory of Slovenia, three areas exhibited the highest probability: one in the west, one in the southwest and one in the southeast (Figure 3). Additionally, large variations in the occurrence of the tree of heaven were observed in the cities of Ljubljana (central Slovenia) and Maribor (northwest Slovenia). The forest model yielded a relatively similar outcome, with the distinction that the probability of occurrence was higher in the northwestern and eastern parts of Slovenia. The model for the tree of heaven for the entirety of Slovenia predicted a lower probability, while the forest model predicted an equal probability from low to high for all cells (Figure 2A).

## 3. Discussion

Our study revealed both overlaps and differences in the potential habitat suitability between American pokeweed and the tree of heaven. In the models for the entirety of Slovenia, both species exhibited a preference for lower altitudes and proximity to water. American pokeweed was predicted to be closer to railways, while the tree of heaven was predicted to be in areas without carbonate bedrock, with land use being a very important variable. In forested areas, the occurrence of American pokeweed is influenced by forest habitat characteristics such as sanitary felling and the presence of Norway spruce monocultures, while the tree of heaven occurrence is primarily influenced by more general environmental variables such as altitude. These findings led to the creation of risk maps that displayed relatively similar patterns for both species, particularly in the case of the tree of heaven. However, the American pokeweed risk map shows many areas, especially forest edges, which are particularly susceptible to invasion. There is also a higher likelihood of the occurrence of this species in areas where sanitation felling has been carried out.

Distance to water bodies was negatively associated with the probability of occurrence for both the tree of heaven and American pokeweed. This could be attributed to the presence of disturbed areas often found in the vicinity of rivers and other waterbodies that provide opportunities for these species to occupy these empty niches (e.g., [33,34]). Furthermore, rivers serve as conduits for the spread of seeds or seedlings of invasive plants [35], and the disposal of garden waste, which often includes alien plant species, is common near rivers.

Alien plant species generally occur at lower altitudes [36,37], and this pattern was also found for American pokeweed and the tree of heaven in our study. Human impacts, including propagule pressure and disturbances, are key factors contributing to the prevalence of alien species in lowland areas. Additionally, studies have demonstrated that the density of roads increases the likelihood of invasion in montane areas [37,38]. This suggests that while the tree of heaven can be found at relatively high altitudes, American pokeweed, which has only recently invaded Slovenia, may have yet to reach higher elevations.

Our study also highlighted the positive effect of the proximity of railway lines on the potential distribution of both species. It is well known that transport routes such as roads, railways and rivers are important conduits for the spread of invasive alien species [39,40,41,42]. The railway network, in particular, has been associated with an increased risk of biological invasions [43,44].

The data on the distribution of the tree of heaven that we used in our study show that the species statistically prefers non-carbonate parent substrate. Interestingly, we also found it on other geologic substrates, especially in the Karst region, which typically has non-carbonate geologic features. In its native China, the tree of heaven is often found in calcareous areas, but it can thrive in a wide range of soil conditions and pH values [45,46]. It is drought-resistant but not flood-tolerant. Čarni et al. [23] found that forest communities thriving on shallow soils over carbonate geological bedrock are at greater risk of being invaded by the tree of heaven in Slovenia. The bias in our presence data towards non-carbonate areas in urban and lowland areas likely contributed to these results.

The tree of heaven is an early-successional species found in forest gaps and clearings [47]. The species therefore is more likely to occur outside the forest where there is more light for its growth. Although it exhibits a competitive life strategy [48], it occurs less frequently in closed forest stands where it is outcompeted by highly competitive late-successional species [49]. Therefore, non-forested areas, including agricultural land, roadsides and railroad lines, urban areas and ruderal sites, provide ideal conditions for the tree of heaven to thrive [50]. Our results regarding land use (e.g., agronomy) as a determining factor for the potential occurrence of the tree of heaven are therefore consistent with its ecology and findings from other studies.

Insolation, which refers to the amount of solar radiation energy received by a given area during a given period, was also a factor influencing the potential spread of the tree of heaven in Slovenia. In addition to anthropogenic factors, which include various transport routes, climatic factors also play a significant role in the invasion of the tree of heaven. Motti et al. [42] found that the distribution of the tree of heaven correlates positively with mean annual temperature as well as temperature during the warmest and coldest quarters.

On a global scale, the tree of heaven is a widespread invasive species in the Northern Hemisphere, adapting to a wide range of climatic conditions, although it is more frequent and abundant in temperate and Mediterranean climates [51]. Cabra-Rivas et al. [52] found that the tree of heaven favours intermediate temperature conditions, with the mean annual temperature being the most influential variable. Motti et al. [42] showed that the tree of heaven favours mild climatic conditions, with low temperatures limiting its distribution at high altitudes.

Interestingly, only American pokeweed was affected by forest stand characteristics. We found that sites disturbed by sanitation felling were more likely to host American pokeweed. The primary mode of natural dispersion for this species is through its fruit, which is consumed by birds [53]. Birds flying over open forest areas created by sanitary felling can deposit seeds of American pokeweed in distant and isolated regions of the country. In these open areas, competition from other plants is relatively low due to the disturbance of the topsoil caused by management interventions, facilitating the spread of this invasive species. Additionally, it was observed that American pokeweed can also occur in relatively shady areas of the forest [32], but in those cases, open areas with adult plants were typically nearby.

Surprisingly, American pokeweed occurred in stands with a higher density of Norway spruce. This might be because both species prefer acidic soil [54]. However, it should be mentioned that not all forest types were sampled with the same intensity, and the forests primarily sampled were in lowland areas. It is possible that as the invasion process continues, this species may also invade other forest vegetation types.

In contrast, the tree of heaven did not show any influence of forest management activities on its probability of occurrence, and only climatic and topographical variables were included in the model. This observation aligns with the fact that this species is a pioneer species that thrives in open, disturbed and extreme habitats [49,55]. Therefore, the inclusion of forest stand factors should only be considered for certain invasive alien species.

Large-scale risk maps typically rely on large-scale databases such as CORINE and include factors with very broad classes. However, our study highlights the need for more detailed factors in forested habitats to determine the risk areas for both selected species. It was demonstrated that models incorporating forest management variables had a higher probability of predicting the presence of American pokeweed. This difference in biology between the two species means that the likelihood of American pokeweed occurring in forests is influenced more by the variables included in the forest model compared to the tree of heaven. Tree of heaven is more of a pioneer species, whereas American pokeweed can also thrive in shaded environments. Additionally, our findings indicated that the forest model for the tree of heaven performed slightly better, with a higher prediction of high occurrence probability than the model for the entirety of Slovenia, even though it did not include specific forest management variables. This difference may be attributable to the increased accuracy resulting from modelling in a smaller and more restricted area.

### Management Implications

Risk maps are an important tool for planning conservation practices, especially for the management of invasive alien species. These maps are instrumental in predicting the potential occurrence and spread of these species. Despite their importance, it is important to consider several shortcomings associated with risk maps.

Our results indicate that relying solely on climate and land use variables may overlook important factors that can be used to predict occurrence on a smaller scale. For example, American pokeweed tends to thrive in canopy gaps in forests and open areas created by large-scale disturbances [27]. Such forest disturbance data are not available in global open databases such as the CORINE database. When preparing a risk map on a national level, it is advisable to use forestry databases that include information on disturbances, with proxies such as sanitation felling. However, in recent years, remote sensing has become increasingly important for detecting large-scale disturbances [56,57,58].

It is also important to take into account the biology of the species. In our study, we created maps for two species, both highly invasive but with distinct biological characteristics. This has implications for the preparation of models and subsequently for risk maps. The tree of heaven, for instance, is more of a pioneer species that typically occupies open areas, whereas American pokeweed is dispersed over larger distances and can thrive in shaded locations. Therefore, different variables were selected when constructing the models. Additionally, the focus on smaller areas or particular habitats, such as forest habitats, in this case, is crucial. We demonstrated that different variables come into play when focussing only on forested habitats, leading to improved predictive accuracy and model effectiveness. We, therefore, recommend tailoring risk maps to the specific habitat types requiring management rather than relying solely on general predictions for larger areas, as this may result in overlooking small-scale distributions.

In conclusion, we have developed habitat models and risk maps for two highly invasive plant species in Europe. Our findings underscore the importance of targeting specific habitat types, as this approach selects different habitat variables and increases the accuracy of predicting the presence of species within those habitats. By increasing the precision of risk maps, the potential to detect these species will increase and, therefore, increase the chances of eradicating or controlling them as early as possible.

## 4. Materials and Methods

### 4.1. Area Description

The study encompasses the entire area of Slovenia (20,273 km^2^). It is one of the most forested countries in Europe, with approximately 58% forest coverage. Slovenia is situated at the confluence of several biogeographic regions: sub-Mediterranean, Alpine, Pre-Alpine, Sub-Pannonian, Pre-Dinaric and Dinaric. The most common tree species in Slovenia are European beech (*Fagus sylvatica* L.), Norway spruce (*Picea abies* (L.) H. Karst) and European silver fir (*Abies alba* Mill.) [59].

In lowland areas that experience periodic flooding, forests occur in narrow strips along rivers and streams and are mainly characterised by willows (*Salix* sp.), alders (*Alnus glutinosa* (L.) Gaertn., *A. incana* (L.) Moench), ashes (*Fraxinus excelsior* L., *F. angustifolia* Vahl) and pedunculate oak (*Quercus robur* L.), with an admixture of European hornbeam (*Carpinus betulus* L.). In hilly areas above the floodplains, mixed forests of sessile oak (*Quercus petraea* (Matt.) Liebl.) and European hornbeam are the predominant forest type. Most of the mid-altitude mountain areas are covered by forests dominated by European beech, with admixtures of other broadleaves (e.g., *Acer pseudoplatanus* L., *Fraxinus excelsior*, *Ulmus glabra* Huds.) and conifers (*Abies alba* Mill., *Picea abies* (L.) Karst.). In the Alpine region, various European beech forests mixed with Norway spruce, European silver fir and European larch (*Larix decidua* Mill.) reach the timberline up to the belt of the dwarf mountain pine (*Pinus mugo* Turra). Scots pine (*Pinus sylvestris* L.) forests occur throughout the country on shallow soils on dolomite and also on acidic, nutrient-poor soils. Minor areas of Austrian pine (*Pinus nigra* Arnold) forests grow on extreme sites with warmer microclimates. The sub-Mediterranean region is covered by forests and shrubby vegetation of thermophilous broadleaf species (e.g., *Ostrya carpinifolia* Scop., *Fraxinus ornus* L., *Sorbus aria* (L.) Crantz, *Quercus cerris* L., *Q. pubescens* Willd.) and Austrian pine *Pinus nigra*. A similar forest type can also be found all over the country on sun-exposed, south-facing slopes with predominant limestone and dolomite bedrock. These natural forest types are also interspersed with secondary forest communities dominated by Norway spruce, which have been significantly affected by climate change and various disturbances in recent decades, such as ice break, windthrows and outbreaks of diseases and pests [17,60]. Forest management in Slovenia follows close-to-nature, sustainable and multifunctional principles, simulating natural processes.

### 4.2. Databases

To create risk maps for the species *A. altissima* and *P. americana*, we considered 11 explanatory variables known to influence the presence of these two species (Table 1). All variables for the analysis and creation of the maps were selected from 1 × 1 km^2^ grid cells and included soil type, geological structure, altitude (m), location or exposure (°), forest association (basic phytosociological unit), larger areas after natural disturbances (total volume of sanitary logging in m^3^), percentage of spruce in the forest stand (%), area of different land use types (m^2^), distance from infrastructure (km), annual precipitation (mm), average annual air temperature (°C) and forest cover (%). The percentage of spruce was included because based on experience, it was found that in planted or unnatural pure spruce stands (monocultures) that are mainly in lower areas in Slovenia, *P. americana* is often found. Furthermore, after sanitary felling of spruce monocultures or spruce-dominated stands of spruce due to drought stress and bark beetles, the spread of *P. americana* was repeatedly observed in these open stands (more light). These open stands have relatively acidified upper layers (horizons) of the soil due to the needles. Probably, in addition to light and acidic soil, other factors are also favourable for the spread, such as disturbance of the soil due to harvesting, and introduction of the species by mechanisation. We used databases from the portal of the Ministry of Agriculture, Forestry and Food (MKGP), the Slovenia Forest Service database of forest stands [61], the cadastre database of economic public infrastructure and the SloClim database [62]. The dependent variables are the occurrence records of *A. altissima* and *P. americana*, which were obtained from the portal Invazivke.si [63]. The portal Invazivke.si was developed in the project LIFE ARTEMIS, which ran from 2016 to 2000 and had the aim of developing an early warning and rapid response framework for Slovenian forests (Table 3). During the project, special attention was given to obtaining observations from citizen scientists on *A. altissima* and *P. americana*. 

### 4.3. Analysis

We developed two models for each species, *P. americana* and *A. altissima*, one for the entire territory of Slovenia and one specifically for forested areas.

A generalised linear model (GLM) with a binomial error structure was used for the analysis. The dependent variables of the model were the presence/constrained pseudo-absence of the selected species. During the modelling process, constrained pseudo-absences were used. In our case, these refer to grid cells visited by observers where only other alien species (not *P. americana* or *A. altissima*) were found [77]. Because there were many more cells with constrained pseudo-absences than cells with the presence of the selected species, an equal number of cells with constrained pseudo-absence data were randomly selected to match the cells in which the selected species occurred.

For American pokeweed, a total of 1384 observations were recorded in 287 cells across the whole of Slovenia. We used a balanced dataset of 574 grid cells, comprising 287 presence and 287 absence data points. In the forest model of American pokeweed, 370 grid cells were used, with 185 cells for presence data and 185 cells for absence data. The tree of heaven was found in 1075 locations in 303 grid cells. A balanced dataset of 606 grid cells, with 303 presence and 303 absence data points was used. For the forest model of the tree of heaven, 318 grid cells were used, with 159 cells for presence data and 159 for absence data. We prepared a training and validation dataset with an 80:20 ratio to perform cross-validation.

Variables were checked for outliers and multicollinearity by visual inspection [78] and the variance inflation factor using the “car” library (version 3.1-2) [79] in the R statistical program (version 4.2.1) [80]. We found that there were strong correlations between altitude, slope, temperature and precipitation. Therefore, we omitted slope, temperature and precipitation due to the detection of strong multicollinearity. Variables were visualised using a density plot prepared in the “ggplot2” library (version 3.4.4) [81].

The model selection proceeded as follows: Initially, we constructed a model with all environmental variables as described in Table 1. The model selection was based on the Akaike Information Criterion (AIC) [82]. The models within four AIC units of the best model were averaged. Subsequently, the validation dataset was used to fit the mean model for validation purposes. Based on these results, we calculated the area under the curve (AUC) in the receiver operating characteristic (ROC) plot, which was calculated for the validation dataset with the “PresenceAbsence” library (version 1.1.11) [83].

## Figures and Tables

**Table 1:** Model statistics for American pokeweed and the tree of heaven across the entire territory of Slovenia and within forested areas.

Species	Area Covered	Variables	Estimate	Std. Error	Adjusted SE	z Value	Pr (>|z|)
*Phytolacca americana*	Entire territory of Slovenia	(Intercept)	3.11	2.19	2.20	1.417	0.156
		Factor (land use)—(agronomy)	1.12 × 10^−1^	1.27	1.27	0.088	0.93
		Factor (land use)—(forest)	8.69 × 10^−1^	1.27	1.27	0.685	0.493
		Factor (land use)—urban area	1.87	1.40	1.41	1.329	0.184
		Mean altitude	−5.66 × 10^−3^	8.44 × 10^−4^	8.46 × 10^−4^	6.688	<2 × 10^−16^
		Distance to water	−1.31 × 10^−4^	5.48 × 10^−5^	5.49 × 10^−5^	2.377	0.0175
		Distance to railroad	−1.10 × 10^−4^	3.20 × 10^−5^	3.21 × 10^−5^	3.421	0.000623
		Factor (geology)—non-carbonate	2.69 × 10^−1^	2.43 × 10^−1^	2.43 × 10^−1^	1.108	0.268
		Insulation	−5.56 × 10^−4^	5.21 × 10^−4^	5.22 × 10^−4^	1.066	0.286
		Distance to roads	−7.86 × 10^−5^	9.08 × 10^−4^	9.11 × 10^−4^	0.086	0.931
	Forested area	(Intercept)	3.84	1.58	1.58	2.427	0.015
		Factor (spruce monoculture)—YES	1.05	3.44 × 10^−1^	3.45 × 10^−1^	3.041	0.002
		Factor (geology)—non-carbonate	7.37 × 10^−1^	3.47 × 10^−1^	3.48 × 10^−1^	2.118	0.034
		Factor (disturbances)—YES	1.32	4.12 × 10^−1^	4.14 × 10^−1^	3.182	0.001
		Mean altitude	−8.09 × 10^−3^	1.27 × 10^−3^	1.27 × 10^−3^	6.363	<2 × 10^−16^
		Distance to water	−1.46 × 10^−4^	7.32 × 10^−5^	7.35 × 10^−5^	1.983	0.047
		Distance to railroad	−1.31 × 10^−4^	4.17 × 10^−5^	4.19 × 10^−5^	3.117	0.002
		Insulation	−3.73 × 10^−4^	6.59 × 10^−4^	6.62 × 10^−4^	0.563	0.573
		Distance to road	−1.08 × 10^−3^	1.86 × 10^−3^	1.86 × 10^−3^	0.577	0.564
*Ailanthus altissima*	Entire territory of Slovenia	(Intercept)	1.79 × 10^−1^	2.80	2.80	0.064	0.949
		Distance to road	−2.28 × 10^−3^	1.24 × 10^−3^	1.24 × 10^−3^	1.843	0.065
		Factor (geology)—non-carbonate	6.17 × 10^−1^	2.55 × 10^−1^	2.56 × 10^−1^	2.413	0.016
		Factor (land use)—(agronomy)	−4.66	2.08	2.09	2.232	0.026
		Factor (land use)—(forest)	−3.52	2.05	2.05	1.717	0.086
		Factor (land use)—urban area	−1.21	2.15	2.15	0.564	0.573
		Insulation	1.34 × 10^−3^	4.36 × 10^−4^	4.37 × 10^−4^	3.066	0.002
		Mean altitude	−4.70 × 10^−3^	7.76 × 10^−4^	7.78 × 10^−4^	6.045	<2 × 10^−16^
		Distance to water	−1.32 × 10^−4^	4.98 × 10^−5^	4.99 × 10^−5^	2.64	0.008
		Distance to railroad	4.14 × 10^−5^	2.37 × 10^−5^	2.38 × 10^−5^	1.742	0.081
	Forested area	(Intercept)	1.76	1.26	1.26	1.398	0.162
		Factor (disturbances)—YES	6.38 × 10^−1^	3.82 × 10^−1^	3.84 × 10^−1^	1.661	0.097
		Mean altitude	−5.54 × 10^−3^	9.04 × 10^−4^	9.08 × 10^−4^	6.096	<2 × 10^−16^
		Distance to railroad	7.97 × 10^−5^	2.74 × 10^−5^	2.75 × 10^−5^	2.897	0.004
		Insulation	3.92 × 10^−4^	4.23 × 10^−4^	4.25 × 10^−4^	0.923	0.356
		Distance to water	−6.21 × 10^−5^	5.87 × 10^−5^	5.89 × 10^−5^	1.055	0.292
		Factor (spruce monoculture)—YES	−2.42 × 10^−1^	3.38 × 10^−1^	3.40 × 10^−1^	0.713	0.476
		Factor (geology)—non-carbonate	2.22 × 10^−1^	3.19 × 10^−1^	3.21 × 10^−1^	0.693	0.488
		Distance to road	−3.35 × 10^−4^	1.66 × 10^−3^	1.67 × 10^−3^	0.201	0.841

**Table 2:** Validation indicators for the models of the entire territory of Slovenia and within forested areas for American pokeweed and the tree of heaven.

Validation Indicators	American Pokeweed		Tree of Heaven	
	Entire Territory of Slovenia	Forested Area	Entire Territory of Slovenia	Forested Area
threshold	0.5	0.5	0.5	0.5
PCC	0.711	0.689	0.727	0.794
sensitivity	0.742	0.743	0.705	0.929
specificity	0.667	0.641	0.75	0.686
Kappa	0.408	0.381	0.455	0.595
AUC	0.773	0.737	0.818	0.839

**Table 3:** Variables used for the creation of risk maps and their databases.

Factor	Databases (Accessed on 10 December 2023)	Unit
Soil type	MKGP Portal, 2007 (https://rkg.gov.si/vstop/) [64]	/
Geological structure	GeoZS: https://ogk100.geo-zs.si/ [65]	Scale: 1:100.000
Altitude	LiDAR: http://gis.arso.gov.si/evode/profile.aspx?id=atlas_voda_Lidar@Arso&culture=en-US [66]	m asl
Exposition	Calculated from DEM (res: 12.5 m) [67]	degrees (°)
Phytocenosis	[68,69]	Scale: 1:100.000
Sanitation felling (proxy for large-scale abiotic and biotic disturbances)	Slovenia Forest Service database, 2021 [70]	m^3^
Spruce monoculture (>70% of all tree species)	Slovenia Forest Service database; forest stand map, 2021 [71]	0 = <70% Spruce; 1 = >70% Spruce
Land use types	MKGP Portal, 2022 (https://rkg.gov.si/vstop/) [72]	m^2^
Distance to infrastructure (roads, railways)	Cadastre of economic public infrastructure, 2022 [73]	km
Annual precipitation	SloClim, 2021 [62]	mm
Annual temperature	SloClim, 2021 [62]	°C
% forest	Slovenia Forest Service database; forest stand map, 2021 [71]	%
American pokeweed occurrence data	www.invazivke.si; [74]	coordinates
Tree of heaven occurrence data	www.invazivke.si; [75]	coordinates
Other citizen science data	www.invazivke.si; from same authors who found American pokeweed and tree of heaven	coordinates
Insolation	[76]	MJ/m^2^

## Data Availability

The dataset is available upon request from the authors.
